# Evaluation of clinical and laboratory findings in severe group COVID-19 pregnants without comorbidity

**DOI:** 10.3906/sag-2105-116

**Published:** 2021-05-10

**Authors:** Fatma BOZKURT, Ömer COŞKUN, Sevda YELEÇ, Muhammed BEKÇİBAŞI, Muhammet ASENA, İhsan BAĞLI

**Affiliations:** 1 Department of Infectious Diseases and Clinical Microbiology, Faculty of Medicine, University of Health Science, Gazi Yaşargil Education and Research Hospital, Diyarbakır, Turkey; 2 Department of Obstetrics and Gynecology, Faculty of Medicine, University of Health Science, Gazi Yaşargil Education and Research Hospital, Diyarbakır, Turkey; 3 Department of Infectious Diseases and Clinical Microbiology, Faculty of Medicine, Bismil State Hospital, Diyarbakır, Turkey; 4 Department of Child Health And Diseases, Faculty of Medicine, University of Health Science, Gazi Yaşargil Education and Research Hospital, Diyarbakır, Turkey

**Keywords:** COVID-19, pregnancy, ferritin, severe disease, 3rd trimester

## Abstract

**Background/aim:**

COVID - 19 disease may be seen with different clinical presentations in pregnant women. Comorbid diseases are important factors affecting the progression of this disease. In this study, we aimed to evaluate the clinical and laboratory findings in pregnant women with COVID - 19 who had no comorbid disease.

**Material and methods:**

This retrospective designed study included 217 patients with Covid PCR positive in typically COVID - 19 clinic. The patients were classified into asymptomatic, nonsevere, and severe disease groups. The symptoms, laboratory results, hospital follow-ups and intensive care records of the patients and the findings of new borns are presented.

**Results:**

Most of the patients (78%) were in the third trimester of pregnancy, and 103 patients in the study group had severe disease. Fever in the non-severe group and respiratory distress in the severe group were the most common symptoms in the patients. The severe clinical manifestations were specifically observed in the third trimester patients. In the severe group, neutrophil, lactat dehydrogenase, ferritin, CK - MB, IL – 6, and hospital stay were statistically higher than those in other groups (p < 0.05). Increase in BUN and creatine were the most predictive parameters in intensive care admission. While the intensive care unit (ICU) requirement was higher in patients in the severe group, premature birth was observed more frequently in the severe group (p < 0.05).

**Conclusion:**

COVID - 19 positive pregnant women were mostly detected in the third trimester, and the disease was more severe in this period. Also, the need for intensive care, the rate of delivery by caesarean section, and the rate of preterm delivery of these pregnant women were also found to be high.

## 1. Introduction

COVID - 19, a disease caused by SARS-CoV-2, is highly infectious and induces death globally. Based on early reports in pregnant women, the clinical course of COVID - 19 is typically mild (86%), severe (9%) or critical (5%), which is like the clinical course distribution seen in the non-pregnant population [1].

SARS - CoV - 2 infections seem to cause serious pulmonary manifestations, including pneumonia [2], acute respiratory distress syndrome, pervasive microemboli, and coagulation perturbations [3]. Therefore, an increase in morbidity and mortality among pregnant women is a reasonable concern. Physiological changes such as hypercoagulation, alterations in cell - mediated immunity [4], decreased lung capacity and pulmonary secretion clearance [5] may increase both the susceptibility and clinical severity of pneumonia in pregnant women. However, common symptoms in pregnancy, such as physiological dyspnea, may delay a diagnosis when pathologic dyspnoea secondary to COVID - 19 is not distinguished [6]. Comorbid diseases, such as diabetes, hypertension, arrhythmia, gestational diabetes and hypertension and preeclampsia, increase morbidity and mortality. However, it could not be differentiated whether the current situation was due to the clinical course of COVID - 19 disease or the underlying comorbid condition [7].

This study aims to examine the clinical course caused by this viral infection, laboratory findings, the need for intensive care, morbidity and mortality rates in COVID - 19 positive pregnant women without comorbid conditions according to the severity of the disease. We also aim to reveal the possible differences between clinical and laboratory findings that occur according to the severity of the disease.

## 2. Materials and methods

This retrospective study included pregnant women who were diagnosed with COVID -19 in a tertiary medical centre between 1 March and 30 November 2020. The study was approved by the Local Ethics Committee (No: 594).

In our clinic, PCR test and thorax computed tomography were evaluated when the pregnant women were admitted to us with fever, flu - like symptoms (headache, pain and burning sensation in the throat and eyes, nasal congestion, muscle and joint pain), respiratory symptoms (cough, shortness of breath and chest pain), gastrointestinal symptoms (nausea-vomiting and diarrhoea) and loss of smell and / or taste. In addition, the COVID - 19 PCR test was performed on patients with a history of contact with a COVID - 19 positive patient, or who were interned for obstetric reasons. Patients with two positive COVID - 19 PCR tests at 24–48 h intervals or patients with a negative COVID-19 PCR test but with typical COVID - 19 compatible thoracic computed tomography (CT) findings were considered COVID - 19 positive and included in the study. Pregnant women with comorbid diseases, such as obesity, diabetes, hypertension, arrhythmia, gestational diabetes and hypertension, gestational cholestasis, preeclampsia, atypical HELLP and chronic liver disease, were excluded from the study (Figure).

In this study, clinical and laboratory findings at the time of admission to the hospital and clinical and laboratory findings at the time of admission to the intensive care unit (ICU) in patients who needed to be followed up in the ICU were recorded as data. The patients’ time interval between symptom onset and admission, vital signs, oxygen saturation, gestational age, gestational week, delivery mode, COVID - 19 -related symptoms with white blood cell (WBC) count, D - Dimer, liver and kidney function markers, cardiac panel (CK-MB, Troponin - I), inflammatory parameters and thorax CT findings were recorded. Clinical and laboratory findings were evaluated separately at the hospitalisation of the ICU. In addition, two consecutive COVID - 19 PCR test results obtained from newborns at the time of delivery and within 24 – 48 hours, alongside the fifth minute APGAR score and birth weight records, were examined. The thorax CTs of the patients were evaluated by two experienced radiologists, and they were classified into 4 grades: early, progression, pike and resolution [8]. According to the classification made by the World Health Organisation (WHO), according to clinical findings and thorax CT results, the disease of the patient was grouped as mild, moderate, severe and critical stages [9]. Mild and moderate patients were regrouped as nonsevere. Since critically ill patients were followed in the ICU at the time of admission, these patients were excluded in the study. In addition to these groups, patients with COVID - 19 without any symptoms were included in the asymptomatic group. Thus, three groups were formed for the study: asymptomatic, nonsevere, and severe. The clinical and laboratory findings of the patients were compared according to the classifications made. Also, the clinical and laboratory findings of the patients taken to the ICU and those requiring no intensive care were also compared.

### 2.1. Statistical analysis

Statistical analysis was performed using SPSS version 22 (IBM, Armonk, NY, USA). The normality of distribution was analysed using Kolmogorov – Smirnov test. Categorical data were expressed as numbers and percentages, and the chi-square test was used for comparison. The Mann – Whitney U test was used for comparison between two independent groups, while the comparison for more than two independent groups was made with the Kruskal – Wallis test. If statistically significant results were observed in the Kruskal – Wallis test, the Mann – Whitney U test was performed between the groups for post-hoc analysis. The values less than 0.05 were considered statistically significant.

## 3. Results

The data of 217 out of 293 COVID - 19 positive patients meeting the criteria were examined; 55 (25.3%) of the patients were in the asymptomatic group, 59 (27.2%) in the nonsevere group and 103 (47.5%) in the severe group (Table 1). Also, 7.4% of the patients were in the first trimester, 13.8% in the second trimester, and 78.8% in the third trimester (Table 1).

There were statistically significant differences when the clinical symptoms and chest CT findings of symptomatic patients were compared. The duration of symptom presence was longer before admission to the hospital in the severe group, and the rate of admission with respiratory tract and GIS symptoms and signs was higher, while the rate of presentation with fever, flu - like symptoms and loss of taste sensation was higher in the nonsevere group (p < 0.05) (Table 1). Radiologically, only two of the involvements were unilateral, while the others were bilateral. Consistent with clinical staging, the rate of the most frequent progression stage was higher in the severe group, while the rate of early stage was higher in the nonsevere group (Table 1).

During hospitalisation, 94/217 (43.3%) of births occurred, and 55/94 (58.5%) of them were caesarean section, 39/94 (41.5%) were vaginally, and 39.4% of them were preterm. Although the rate of caesarean delivery in the severe group exceeded those of the other two groups, there was no statistically significant difference (p > 0.05). The rate of preterm labour was higher in the severe group compared to the other two groups, and there was a statistically significant difference (p < 0.05). While maternal hypoxemia and fetal distress constituted the caesarean indication in the severe group, the difference was statistically significant in the other two groups (p < 0.05) (Table 2). In the COVID -19 PCR scans of 92 newborns, the first COVID - 19 RT – PCR test taken immediately one baby was born was positive (the APGAR score was 9, and the girl was 3450 gr.). Significant differences were found between the groups regarding length of hospital stay, aspartate aminotransferase (AST), alanine aminotransferase (ALT), D - DIMER, C -reactive protein (CRP), neutrophil (NEU), lactate dehydrogenase (LDH), ferritin, creatine kinase MB (CK - MB), interleukin - 6 (IL - 6), lymphocyte, eosinophil and albumin levels (p < 0.05) (Table 3).

During hospitalisation, 37 patients (4 asymptomatic, 7 non - severe and 26 severe groups) were followed up in the ICU for approximately 4 days (2 – 6) due to worsening in breathing; 33 out of 37 patients (89.2%) entering the ICU were 3rd trimester patients (Table 4). Thorax CT of eighteen patients taken during admission to intensive care were compatible with progression and 19 with peak stage. Twenty of the patients were given high flow oxygen (HFO), and 17 of them were taken to mechanical ventilation support, three of which were invasive. During the hospital follow-up period, 10 / 37 (27%) of the patients followed in the ICU did not give birth, while 73% (17 caesarean and 10 vaginal deliveries) were delivered. A total of 22.2% of those who gave birth (6 / 27) were taken to the prenatal care unit, and 21 / 27 (77.8%) were taken to the postnatal intensive care unit. Duration of symptoms before hospitalisation, body temperature, systolic blood pressure, pulse, respiratory rate, neutrophil percentage, blood ure-nitrogen (BUN), creatine (CREA), AST, ALT, LDH, CK, D - Dimer, Ferritin, CRP, CK - MB, Tro I and IL - 6 median were high; the 5 - min APGAR score, SPO2, WBC, lymphocyte and eosinophil percentage and ALB median were low, and the difference was statistically significant (p < 0.05) (Tables 4 and 5).

In total, four of our patients who were admitted to ICU were complicated with postpartum pulmonary embolism and one patient with prenatal myocarditis. There was no maternal mortality during hospital follow-up and treatment.

## 4. Discussion

In this study, COVID - 19 RT - PCR test positivity was 92.6%, and 47.5% of pregnant women were included in the severe group. This study is imperative because it is a comprehensive study conducted in a single centre, including patients with similar demographic characteristics.

Jie Yan et al. [10] identified fever in 50.9%, cough in 28.4%, and dyspnoea in 7.8% of a group of pregnant women, and only 6.9% of them were severe. Delahoy et al. [11] analysed the surveillance data of 598 pregnant women and identified fever in 59.6% and coughing in 59.2%. Mohr Sasson et al. [12] reported that 54.5% of 11 pregnant women showed respiratory symptoms, 27% had fever, and respiratory symptoms were more common than fever. However, in the above studies, admission symptoms were not evaluated following clinical classification. Furthermore, Pierce-Williams et al. [7] reported application clinical symptoms of 44 severe and 20 critical pregnant women and reported an average of 7 days between the onset of the symptoms and hospital application.

Typical symptoms of COVID - 19 are fever and respiratory symptoms, while atypical symptoms include GIS (abdominal pain, diarrhoea and nausea-vomiting) and symptoms and signs of neurological involvement [13]. This was an important determination regarding the fact that fever, which is the typical symptom of COVID-19 during the pandemic period, was not in pregnant admissions, indicating that the clinic could be severe. The absence of fever in pregnant women in the severe group can be explained by the increase in immune suppression and decrease in fever response in parallel with the increase in the severity of the disease in pregnant women who are immunosuppressive [4,5].

In a multicenter study that included 355 COVID - 19 patients, 39.7% of patients were taken to the ICU. It was emphasised that dyspnoea, tachypnoea and low peripheral oxygen saturation are important parameters in showing the severity of the disease, and they might be an indicator to predict intensive care requirement [14]. A single - centre study revealed that data such as heart rate, respiratory rate, and mean arterial pressure did not differ in patients admitted to the ICU, and the patient required no intensive care [15].

In a systematic review and meta - analysis of 4062 COVID - 19 positive non-pregnant adult cases, it was reported that tachypnoea induced by the decrease in oxygen saturation in critical patients was a discriminative finding; heart rate did not differ between the severe and non - severe groups [16,17], and blood pressure was reported higher in the severe group in a study [16], while it was not in another study [17]. In our study, like non - pregnant adults, in pregnant women, there were no significant differences in blood pressure and heart rate during application among groups, but the increased respiratory rate and low oxygen saturation in the severe group at presentation were found to be statistically significant compared to the other two groups [14,17]. In our study, we observed that heart rate and blood pressure were similar between the groups, but respiratory rate and oxygen saturation were significantly altered according to the severity of the disease. Increased respiratory rate and low oxygen saturation can be important indicators to distinguish physiological dyspnoea seen in pregnant women, especially in the third trimester, and dyspnoea seen in COVID - 19 positive pregnant women.

Yuming et al. [18] reported that the main laboratory findings were lymphopenia and elevated CRP in 146 pregnant women, 5.5% of which were severe. In their research, Scott et al. [19] studied 69 COVID - 19 positive pregnant women, 15 of which required respiratory support, and they reported that the lymphocyte rate was lower in those who required respiratory support; however, WBC, neutrophil and PLT rates were similar. CRP was elevated in 63 patients during hospitalisation; however, there was no statistically significant difference between the groups. Andrikopoulou et al. [20] reported significantly elevated ferritin and leucopenia levels in moderate or severe disease groups, while the differences between ALT, AST, LDH, IL - 6, CREA, platelets, lymphocyte and D -dimer levels were reported not statistically insignificant.

In our study, hematological tests of asymptomatic, non-severe and severe group patients were compared concurrently. On admission to the hospital, increased LDH, CRP and IL - 6 and low albumin were found to be predictive parameters in distinguishing the asymptomatic group from the non-severe group. However, the most distinctive predictive parameter among all three groups was ferritin, and it was found to be the only statistically significant parameter with an increase above the reference range in the severe group. When those taken to the ICU were compared with those who were not, worsening of breathing in those who were taken to the ICU was accompanied by increased heart rate, systolic blood pressure, respiratory rate and decreased oxygen saturation as parameters show a statistically significant difference. It was found statistically significant that the median of lymphocyte and albumin was lower, and NEU, LDH, CRP, D - DIMER and IL - 6 were higher in those who were taken to the ICU but were outside the reference range in both groups. However, ferritin, BUN and CREA, which showed a statistically significant increase above the reference range in the intensive care patients, were found to be predictive parameters. These were important parameters in showing an increase in inflammation and kidney involvement of COVID - 19 in those taken to the ICU.

In a multicenter study in France, 617 COVID - 19 infected pregnant women classified as 79.2% non-severe, 15.2% receiving respiratory support (receiving nasal oxygen and/or mechanical ventilation support) and 5.6% critical group (receiving invasive mechanical ventilation or ECMO) were examined. The severity of the disease was found to be associated with the presence of over 35 years of age and comorbid conditions. In the neonatal nasal COVID - 19 RT - PCR test screening, only two newborns had the test positive. While there was no COVID - 19 - related neonatal death, a mother who was followed in the ICU in the critical form group was lost due to COVID - 19 [21]. Only one patient was found positive in the neonatal nasopharyngeal COVID - 19 RT - PCR scan in our study. This newborn was discharged with recovery with his mother. We lacked enough cases about the transition from mother to baby, but in three different studies presented on this subject, it was stated that the vertical transmission was not at a significant level [22,23]. In the large - series prospective multicenter study in the UK, 427 symptomatic - hospitalised COVID - 19 - infected pregnant women, 81% of whom were in the third trimester, were analysed. A total of 41% of the patients were aged 35 or over, and 46% had comorbid conditions [24].

Contrary to the studies mentioned, the majority of the patients in our study population comprised pregnant women with severe disease, and 78.8% of the cases were third trimester pregnancies according to the trimester distinction. The disease progressed more severely in three trimesters of pregnant women in our study population. 76.7% of the hospitalised patients and 70.2% of those taken to the ICU were severe group, 3rd trimester pregnant women. The reason for the severe course of the disease in pregnant women infected with COVID - 19 can be explained by the physiological changes that occur in the respiratory and immune systems of pregnant women. In the physiological changes that occur in the immune system in pregnant women of third trimesters, Th1 and Th2 cell balance shifts to the Th1 side, and the inflammatory process dominates. This contributes to exaggerated inflammation in the pathophysiology of COVID - 19, inducing a cytokine storm in 3rd trimester pregnant women. In addition, the increased uterine volume in third trimester pregnancies elevates the maternal diaphragm, resulting in a severe course of pneumonia clinic by increasing the sensitivity of respiratory viruses and decreasing the tolerability to hypoxia by increasing the physiological changes in the respiratory system, including the decrease in total respiratory capacity, functional residual capacity, end - expiratory volume and residual volumes of the lung [25].

Following the current literature, 58.5% of the deliveries in the hospital occurred with caesarean section and 39% with preterm labour [21,24]. Maternal hypoxemia and foetal distress constituted 40% of cesarean indication. Similarly, the rates of caesarean section due to caesarean section, preterm labour and maternal hypoxemia or foetal distress were found to be higher, with a statistically significant difference in pregnant women in the severe group and those taken to the ICU. In addition, the APGAR score of the babies of mothers taken to the ICU was found to be statistically significantly lower, attributed to the mother’s illness. However, there was no mother or baby loss because the patient population comprised young and pregnant women without comorbid conditions, with the treatment and follow - up protocol of the patients being conducted by a multidisciplinary team (infectious diseases, gynaecology, anaesthesia and perinatology physicians) in a single centre.

Our study has some limitations. Despite our high-patient admission rates, the records for the data of outpatients and third trimester pregnant women who gave birth in our hospital after discharge were excluded from the study because they could not complete the study criteria. In addition, this evaluation could not be made because records for APACHE and Glaskow Coma Scale were insufficient for patients followed in the ICU. Neonatal vertical transition was evaluated only by nasopharyngeal and/or oropharyngeal sampling. The COVID - 19 PCR study was not performed on respiratory secretions, cord blood, amniotic fluid, vaginal fluid or breast milk samples. However, no sampling was done for postpartum horizontal transition. However, we think that this study is useful regarding monitoring many cases in a single centre.

## 5. Conclusion

In this study, it was determined that COVID - 19 diseases had a more severe course in third trimester pregnant women, and the period between the onset of COVID – 19 -related symptoms and admission to the hospital was long in pregnant women with severe disease. Hence, pregnant women with symptoms in the third trimester should be followed up closely. Contrary to nonpregnant adults, observably, fever is not the main symptom in pregnant women who are physiologically immunosuppressive and present with respiratory symptoms in pregnant women with a severe course of the disease. It was found that dyspnoea is an important symptom in adult patients with a severe course of the disease, but it can be confused with the presence of physiological dyspnoea in pregnant women, and the presence of oxygen saturation and tachypnoea are important parameters in predicting the severity of the disease in pregnant women. The most predictive parameter in showing the disease severity at presentation was the increase in ferritin in pregnant women. The most predictive parameters were the increase in BUN and CREA, in addition to the increase in ferritin in the intensive care entry. The absence of maternal and neonatal loss may be related to the follow-up of many patients in a single centre.

## Figures and Tables

**Figure:**
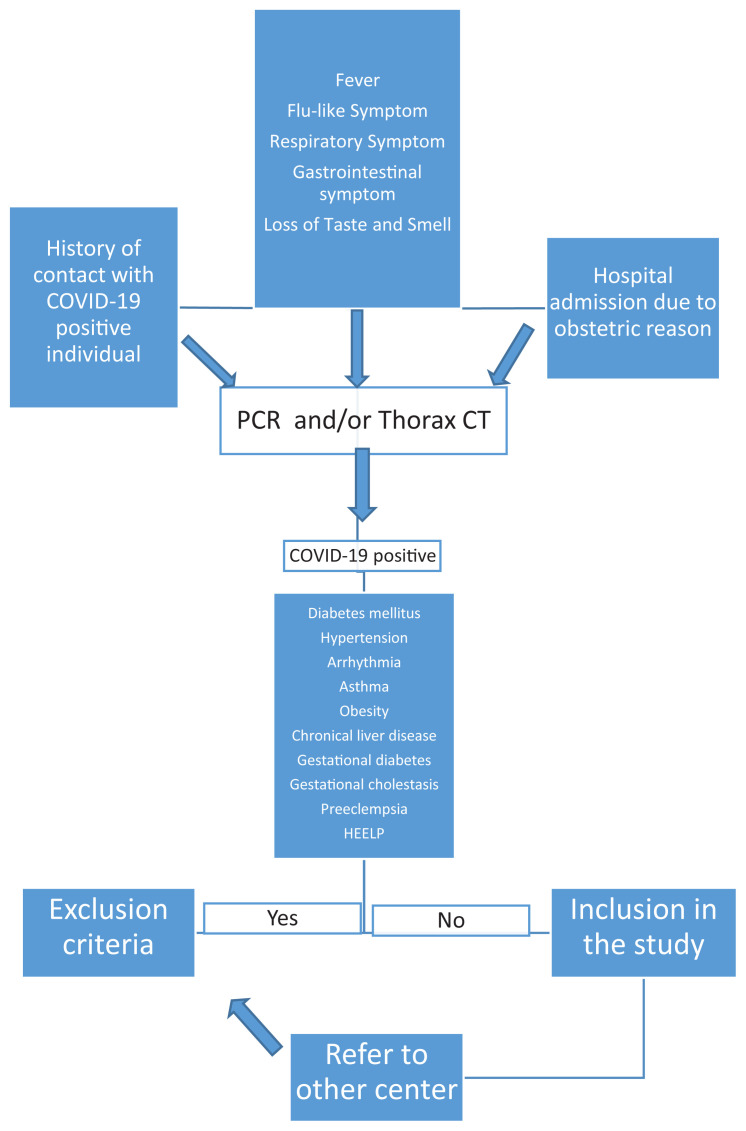
Inclusion and exclusion criterias of the Covid-19 positive pregnant women.

**Table 1:** Clinical characteristics of COVID-19 positive pregnant women according to clinical classification on hospital admission.

	Asymptomatic Median (min-max)/ n (%) (n:55)	Non-severe Median (min-max)/ n (%) (n:59)	Severe Median (min-max)/ n (%) (n:103)	p value
**Age (years)**	28 (17–45)	30 (16–41)	30 (18–44)	0.758
**Symptom onset-admission to the hospital day**	-	7 (2–14)	8 (3–18)	**0.006**
**Trimestr**				0.108
**1**	4 (7.3%)	7 (11.9%)	5 (4.9%)	
**2**	3 (5.5%)	8 (13.6%)	19 (18.4%)	
**3**	48 (87.3%)	44 (74.5%)	70 (67.9%)	
**COVID-19-related symptoms**				
**Fever ( ≥ 38.3 ºC)**	-	31 (52.5% )	11 (10.7%)	**<0.001**
**Flu-like symptoms**	-	33 (55.9%)	4 (3.9%)	**<0.001**
**Respiratory Symptoms**	-	30 (50.8%)	99 (96.1%)	**<0.001**
**Loss of taste and smell**	-	22 (37.3%)	12 (11.7%)	**<0.001**
**GIS Symptoms**	-	7 (11.9%)	22 (21.4%)	**<0.001**
**Chest CT staging**				**<0.001**
**Early**	-	44 (74.6%)	6 (5.8 %)	**<0.001**
**Progression**	-	8 (13.6%)	90 (87.4%)	**<0.001**
**Peak**	-	0	7 (6.8%)	
**No involvement**	-	7 (11.9%)	-	

Note: GIS: Gastrointestinal system; CT: Computed Tomography.

**Table 2:** Delivery characteristics of COVID-19 positive pregnant women according to clinical classification.

	Asymptomatic Median (min-max)/ n (%) (n:55)	Non-severe Median (min-max)/ n(%) (n:59)	Severe Median (min-max)/ n (%) (n:103)	p value
**Delivery mode**				
**C/S**	17 (50%)	8 (44.4%)	30 (71.4%)	0.068
**Vaginal**	17 (50%)	10 (55.6%)	12 (28.6)	
**Preterm birth**	4 (11.8%)	6 (33.3%)	27 (64.3%)	**<0.001**
**Previous C/S**	13 (76.5%)	5 (62.5%)	3 (10%)	**<0.001**
**Other C/S indications**				
**Fetal distress**	1 (2.9%)	-	12 (28.6%)	**<0.001**
**Maternal hypoxia**	-	-	9 (21.4%)	
**Induction failure**	2 (5.9%)	-	4 (9.5%)	
**Malposition**	1 (2.9%)	3 (16.7%)	2 (4.8%)	
**Baby Birth Weight (gr)**	3450 (2780–3200)	3240 (1700–4300)	3120 (1650–3200)	0.308
**5. min APGAR**	9 (6–10)	9 (7–10)	8 (3–9)	0.674
**Hospital days**	3 (1–5)	5 (3–9)	8 (6–16)	**<0.001**

Note: C/S: Cesarean section; APGAR: Appearance, Pulse, Grimace, Activity, and Respiration.

**Tablo 3:** Laboratory findings of COVID-19 positive pregnant women according to clinical classification on hospital admission.

	Asymptomatic (n:55) Median (min-max)/ n (%)	Non-severe (n:59) Median (min-max)/ n (%)	Severe (103) Median (min-max)/ n (%)	p value
Heart rate (n/min)	110 (90–145)	110 (91–146)	112 (90–150)	0.223
Systolic pressure (mm/Hg)	80 (60–98)	81 (62– 99)	87 (64– 100)	0.313
Diastolic pressure (mm/Hg)	90 (77–122)	90 (78–142)	91 (79–142)	0.871
Respiratory rate (n/min)	21 (14–23) β	22 (15–34) γ	33 (19–54)	**< 0.001**
SPO_2_	97 (95–99) β	96 (90– 98) γ	90 (75– 91)	**< 0.001**
WBC (10^9^/L)	9.2 (3.5–15.4) αβ	7.6 (2.2–18.6) γ	6.4(2.2–14.5)	**0.027**
Lymphocytes (%)	17.1(3.9–31.5) αβ	10.2 (2–26.4) γ	7.8(1.1–25.5)	**0.013**
Neutrophils (%)	74.5 (65–94) αβ	80 (62–94) γ	88 (61–99)	**< 0.001**
Eosinophils %)	3.4 (0.1–4.8) β	3.3 (0– 4.4) γ	0.6 (0–3.2)	**< 0.001**
PLT (10^9^ / L)	252 (102–340)	223 (51–355)	218 (42–367)	0.922
BUN (mg/dL)	29 (10– 317)	23 (9 – 238)	26 (8–331)	0.284
Creatinine (mg/dL)	0.63 (0.3– 1.6)	0.7(0.3– 1.67)	0.7(0.3–1.74)	0.310
AST (U/L)	36 (14–228)	35 (16– 242) γ	37 (36– 1265)	**0.010**
ALT (U/L)	19 (8–130) β	18 (8– 133) γ	38 (27–1152)	**0.031**
LDH (U/L)	183 (126–422) αβ	269 (127– 726) γ	330 (138– 2271)	**<0.001**
CK (U/L)	34 (13–296)	64(12 –292)	70 (13– 658)	0.070
ALB (g/L)	40 (30–51) αβ	30 (28– 49)	29 (17– 45)	**< 0.001**
D-Dimer (ng/mL)	240(119–1278) αβ	352(120–1230) γ	527(145–5948)	**0.004**
Ferritin (ng/mL)	26 ( 7–270)	47 ( 9–578) γ	227 ( 12– 762)	**< 0.001**
CRP (mg/L)	6 (2 – 27) αβ	27.8 (11 – 145) γ	72 (15 –271)	**< 0.001**
CK-MB (ng/mL)	0.77 (0.39–8.8) αβ	1.32 (0.55–6) γ	3 (0.56 –7.8)	**< 0.001**
Troponin- I (ng/mL)	0.1 (0.1–0.4) β	0.1 (0.1– 0.6)	0.1 (0.1–0.63)	**0.009**
IL- 6 (ng/L)	7 (4–72)	16 (4.2–101)	56 (5–112)	**< 0.001**

**Note:** SPO_2:_ oxygen saturation; WBC: white blood cell count; PLT: platelet count; BUN: blood ure-nitrogen; ALT: alanine aminotransferase, AST: aspartate aminotransferase; LDH: lactate dehydrogenase; CK: creatine kinase; ALB: albümin; CRP: C-reactive protein; CK-MB: creatine kinase MB, IL-6: Interleukin-6.

α significant difference between asymptomatic and non-severe group;

β significant difference between asymptomatic and severe group;

γ significant difference between non-severe and severe group.

**Table 4:** Clinical characteristics of COVID-19 positive pregnant women according to intensive care unit admission.

	Non-ICU (n:180) Median (min-max)/ n (%)	ICU (n:37) Median (min-max)/ n (%)	P Value
**Age (years)**	29 (16–45)	30 (20–42)	0.355
**Symptom onset-admission to the hospital day**	7 (1– 17)	11 (7–18)	< 0.001
**Clinical Stage**			0.008
**Asymptomatic**	51 (28.3%)	4 (10.8%)	
**Non-severe**	52 (28.9%)	7 (18.9%)	
**Severe**	77 (42.8%)	26 (70.3%)	
**Trimestr**			0.225
**1**	15 (8.3%)	1 (2.7%)	
**2**	27 (15.0%)	3 (8.1%)	
**3**	138 (76.7%)	33 (89.2%)	
**Delivery**			< 0.001
**No**	113 (62.8%)	10 (27.0%)	
**Yes**	67 (37.2%)	27 (73.0%)	
**Delivery mode**			< 0.001
**C/S**	38 (21.1%)	17 (45.9%)	
**Vaginal**	29 (16.1%)	10 (27.0%)	
**C/S indications**			< 0.001
**Previous C/S**	17 (9.4%)	4 (10.8%)	
**Induction failure**	5 (2.8%)	1 (2.7 %)	
**Malposition**	4 (2.2%)	2 (5.4 %)	
**Maternal hypoxia**	4 (2.2%)	5 (13.5%)	
**Fetal distress**	8 (4.4%)	5 (13.5%)	
**PretermLabor**	25 (13.9%)	12 (32.4%)	**0.006**
**BabyBirthWeight (gr)**	3420 (1650–3200)	3120 (1900 –3750)	0.084
**5. min APGAR**	9 (6–10)	7 (3–10)	< 0.001
**Hospital stay (day)**	5 (1–10)	9 (3–16)	< 0.001

Note: ICU: Intensive-care unit; C/S: Cesarean section; APGAR: Appearance, Pulse, Grimace, Activity, and Respiration.

**Table 5:** Laboratory findings of COVID-19 positive pregnant women in ICU admission

	Non- ICU Group (n:180) Median (min-max)/ n(%)	ICU group (n:37) Median (min-max)/ n(%)	P
**Temperature (°C)**	37 (36.5 – 39.3)	37.3 (36.5–39)	**0.021**
**Heart rate (n/min)**	90 (77–134)	119 (81–145)	**0.004**
**Systolic pressure (mm/Hg)**	110 (90–140)	120 ( 98–150)	**0.048**
**Diastolic pressure (mm/Hg)**	80 (60–100)	89 (63–109)	0.051
**Respiratory rate (n/min)**	20 ( 14–34)	26 ( 24–54)	**0.012**
**SPO** **_2_**	95 ( 80–98)	90 ( 75–95)	**< 0.001**
**WBC**	7.6 ( 2.2– 8.6)	7.3 ( 2.3–18.2)	**0.003**
**Lymphocytes**	10.8 ( 3.4–31.5)	8.6 ( 1.1–18.6)	**0.030**
**Neutrophils**	82 ( 61–96)	85 ( 63 – 99)	**0.016**
**Eosinophils**	1.7 ( 0–4.8)	0.6 (0–4.3)	**0.004**
**PLT**	233 (51–369)	223 (43–337)	0.875
**BUN**	23 (8–119)	223 (15–331)	**< 0.001**
**CREA**	0.6 (0.3–1.3)	1.45 (0.34–1.74)	**< 0.001**
**AST**	35 (14–290)	38 (24–1264)	**< 0.001**
**ALT**	19 ( 8–266)	23 (15–1138)	**0.003**
**LDH**	257 ( 126–2224)	321 (134–2265)	**0.010**
**CK**	56 (11–478)	83 (13–647)	**0.021**
**ALB**	33 ( 25–49)	29 (17–40)	**< 0.001**
**D**–Dimer	354 (119–3352)	658 (147–5948)	**< 0.001**
**Ferritin**	68 (9–634)	293 (7–762)	**< 0.001**
**CRP**	31.5 (2–236)	77 (24–271)	**< 0.001**
**CK**–MB	0.99 (0.4–8.8)	2 (0.55–8.89 )	**< 0.001**
**Tro I**	0.1 ( 0.1–0.4)	0.1 (0.1–0.6)	**< 0.001**
**IL 6**	16.2 (4–101)	34 (6.2–105)	**< 0.001**

Note: ICU: Intensive care Unit, SPO_2:_ oxygen saturation; WBC: white blood cell count; PLT: platelet count; BUN: blood ure–nitrogen; ALT: alanine aminotransferase, AST: aspartate aminotransferase; LDH: lactate dehydrogenase; CK: creatine kinase; ALB: albümin; CRP: C–reactive protein; CK-MB: creatine kinase MB, IL-6: Interleukin-6.
